# Ethyl Chloride—Its Advantages and Disadvantages

**Published:** 1906-12-22

**Authors:** 


### SPECIAL ARTICLE.

### ETHYL CHLORIDE—ITS ADVANTAGES AND DISADVANTAGES.

When ethyl chloride was re-introduced to the medical profession in 1898 by Lotheisen, of Vienna, as a general anæsthetic for surgical operations, it was welcomed as being so safe as to be practically devoid of any risk: it was pleasant to take, simple to administer, and, above all, required no elaborate apparatus, and was therefore portable to an extent only comparable with chloroform, whose dangers were so well recognised. It promised, in fact, to displace nitrous oxide from its position as the *safe* anæsthetic; for its administration a qualified medical man was scarcely held to be essential, and it was rapidly adopted by dentists and used in their work with as little compunction as nitrous oxide.

Recent investigations, and particularly the work of McCardie of Birmingham, tend however to show that, in spite of the numerous advantages the drug possesses, such sweeping claims on its behalf are not justified by experience.

### Its Death-Rate.

Notwithstanding the glowing pictures at first drawn of an anæsthetic without a "death-risk," eight or ten years' use of the drug has compelled a slight change of attitude in this particular. Fatalities, few and far between it is true, have been placed on record until we have now perhaps sufficient data from which to calculate its average death-rate, and by which to compare its safety with that of the other anæsthetics in general use. It is now recognised that no less skill and special knowledge is needed for its safe handling than for nitrous oxide, ether, or chloroform. Pathological investigation of the blood in fatal cases has shown a change in the appearance of the blood somewhat similar to that of carbonic acid poisoning. The patients who have died during ethyl chloride anæsthesia have been such as we associate with death during chloroform administration, the subjects of fatty heart, alcoholics with universal degeneration of the organs and blood-vessels, and patients suffering from inflammatory conditions about the jaw or throat; just those whom any cautious anæsthetist would approach with misgivings. This, however, does not agree with the assertions of the earlier advocates of ethyl chloride; Girard, for instance, who maintained that "circulatory troubles and respiratory affections did not contra-indicate its use; neither age, nor sex, nor alcoholism, nor other intoxications forbade its administration. The only doubtful factor was the state of the urinary organs." Lotheisen at first estimated the mortality at 1 in 2,500, but later placed it at 1 in 17,000. Seitz, in about 1902, reckoned it to be 1 in 16,000. McCardie, who has carefully investigated twenty-one fatal cases, places the death-rate as high as 1 in 3,000, which is a striking figure when we remember that the mortality under ether is given as 1 in 16,000, and that of nitrous oxide as 1 in 1,000,000.

### Its Relation to Other Anæsthetics.

From a consideration of the chemical composition of ethyl chloride the toxicity of this drug would be placed below that of chloroform, but above that of ether and therefore of nitrous oxide. Nitrous oxide stands, as regards its mortality, in a class by itself; it appears to be practically safe. Ethyl chloride would appear on McCardie's reckoning to be less safe than ether and far safer than chloroform. This classification is a new one. At the outset claims were made for ethyl chloride which would have encouraged the view that there was far less risk in its administration than in the use of ether; but it now seems that this confidence is not justified by results. Bearing in mind that ethyl chloride is for the most part only used for short operations of a "minor" nature, and that ether is the anæsthetic commonly chosen for long and serious operations, their relative safety on the foregoing estimate must be reversed, and ether regarded as being the freer from risk, though it is neither pleasant to take nor handy to use for anæsthesia of short duration.

Nitrous oxide should probably always be preferred for trivial operations, and ethyl chloride reserved for those intermediate between this class and that in which ether is customarily used.

### Ethyl Chloride for Children.

A striking fact in connection with its mortality is its comparative safety in children. Scarcely any fatalities have been reported except in adults, and for children it has many other advantages. Anæsthesia is much more quickly induced; the inhalation of the vapour is not unpleasant and does not cause a sensation of suffocation; the apparatus and its preparation has nothing to inspire the most timid child with terror (and the part played by fright in chloroform-deaths among children has never been fully recognised, and perhaps is impossible to determine); while, finally, the recovery from the anæsthetic in children is scarcely ever attended by the unpleasant symptoms of nausea and faintness which adults so often experience. Ethyl chloride can be, without hesitation, administered to infants under a year old, even to the weak and ill-developed specimens met with in children's hospitals. Elaborate preparation, so difficult to have properly carried out by the mothers of out-patients, seems not at all essential; and as the recovery period is short and devoid of ill-effects, the children can be sent home much sooner than is possible after chloroform. For the opening of empyema, pharyngeal abscess, removal of tonsils and adenoids, as well as the many minor operations such as circumcision, nævi, etc., it is preferable to any other anæsthetic.

### Ethyl Chloride in Dental Practice.

Of the fatalities collected by McCardie it is noteworthy that a very large proportion of them occurred during administration for dental operations. It is true that the anæsthetic in this work is still given freely by men without a medical or dental qualification; and this increases the need for insisting upon the skill which is required for, and the caution which should attach to, its use. In general, nitrous oxide will serve for nearly all dental operations. They can be performed if necessary, and in many cases far better, at several sittings. Prolonged administration of nitrous oxide through the nose can be easily carried out by means of the special apparatus devised for the purpose, while, apart from any question of possible fatalities under ethyl chloride, the rapid and uneventful recovery from nitrous oxide is essential to the performance of dental operations where the patient is not in his own home; the onset of any untoward after-effects, such as syncope, vomiting, or delayed recovery of consciousness which may follow ethyl chloride anæsthesia being most embarrassing to the dental surgeon if they should occur in his consulting-room.

### Danger Signals.

Under this heading a discussion on "Snakes in Iceland" might be, with some degree of propriety, introduced. There is a remarkable unanimity amongst those observers who have reported fatalities, that of "danger signals" there are none. Whether this is strictly correct is open to some doubt; but, at any rate, in almost every case the warning was terribly short and death instantaneous. Seeing, too, that some of the deaths took place in the practice of anæsthetists of experience, it must be admitted that the "danger signals" associated with ether or chloroform anæsthesia did not present themselves. One striking case reported from the Haslar Hospital will serve to illustrate this. "An able-bodied seaman was anæsthetised with a usual dose of ethyl chloride, and two or three teeth were extracted; the patient recovered sufficiently to expectorate once, and then fell back in the chair—dead!" Again, "A gentleman aged 67 was given ethyl chloride for the extraction of four teeth. He became pale, and died in the dental chair from syncope." And a third case, in which more details are given: "A female aged 18, to whom exactly 5 ccm. of ethyl chloride were administered from an Ormsby inhaler. The patient went under without struggling, and the conjunctival reflex was present just before stertor occurred, at the first sound of which the inhaler was removed and some ether poured in. Before the inhaler was reapplied the patient's respiration became shallow and her pulse impalpable. She breathed several times afterwards, but no cardiac action was noted." These cases serve to make us cautious, but they do not show any means whereby the fatality can be avoided or the danger foreseen—a danger which, as McCardie says, "often appears with lightning-like rapidity." There seems a discouraging absence of premonitory symptoms, which detracts from the administrator's feeling of security usually engendered of knowledge; but here increased familiarity with the drug leads to greater respect for its powers.

Possibly the English "closed" method of administration is responsible for the higher mortality than on the Continent, where a "semi-open" method allowing of a freer admixture of air is adopted; but further statements on these points are most important if ethyl chloride is to be administered with any feeling of certainty on the part of the anæsthetist.

The corneal reflex, the pupil, and the pulse alike seem to fail as indicators of approaching danger.

### After-Effects.

Of these, the most important is collapse: it occurs chiefly after prolonged administration where no fresh air has been allowed; the liability to it is increased by the use of a small bag, and by allowing the bag to become collapsed during administration. It seems probable that the breathing of carbon dioxide plays some, if not the main, part in producing this effect, or perhaps some of the other volatile bodies which are derived from the ethyl chloride and are re-inspired. At all events, the admixture of air with the ethyl chloride seems to prevent this tendency to collapse; and not only this complication, but nausea and headache appear to owe their origin to the same causes, for by similar means they can to a large extent be avoided.

Vomiting, when it does follow the administration of ethyl chloride, may assume a form even more severe than that which is caused by ether or chloroform, and in a few instances may only be checked by the hypodermic injection of morphia. Such cases, fortunately, are very rare. The cause should be looked for elsewhere than in ethyl chloride.

But delay in the recovery of consciousness, excitement, and headache are comparatively frequent, so as to cause regret on many occasions when nitrous oxide was at hand and could have easily been given with advantage not only to the operator and anæsthetist, but, most important of all, to the patient.

### Preliminary to Ether or Chloroform.

In place of nitrous oxide as a preliminary to the other anæsthetics, ethyl chloride has many claims beyond its greater handiness. There is, as a rule, no struggling stage, the patient passes without any tendency to recover consciousness from the first to the second anæsthetic, and the time occupied is distinctly shorter. In the case of chloroform, ethyl chloride may be sprayed on to the open mask, and the chloroform immediately started in the ordinary fashion; with ether the bag of the Clover inhaler may be sprayed with ethyl chloride, and the ether gradually turned on just in the way adopted when nitrous oxide is used as the preliminary anæsthetic. For the rapid anæsthetisation of the alcoholic it seems admirable.

### Conclusion.

Finally then, we may assume that having taken its place not as superior to but among the other anæsthetics, ethyl chloride has come to stay. That in future its chief sphere of usefulness will be amongst children, upon whom minor operations are common, and for whom ethyl chloride has least danger. That as a preliminary to ether and chloroform it has distinct advantages over nitrous oxide, and that for single-handed operations of short duration or for easy transport in an extensive country practice it occupies a position which no other drug has so far made any pretence to fill.

